# On the Treatment of Subacute Bronchitis
1A paper read at a meeting of the Bristol Medico-Chirurgical Society, on February 8th, 1899.


**Published:** 1899-09

**Authors:** F. H. Edgeworth

**Affiliations:** Assistant-Physician to the Bristol Royal Infirmary.


### ON THE TREATMENT OF SUBACUTE BRONCHITIS.[1]

BY

F. H. Edgeworth, M.B., B.Sc., B.A. Cantab.,

*Assistant-Physician to the Bristol Royal Infirmary.*

The following notes deal with the treatment of ordinary subacute bronchitis, of such cases as come in numbers to the out-patient department of a hospital and are found so frequently in medical practice. They do not touch on questions of complications and sequelæ, or on the methods that may be adopted in severe cases of the disease.

[1] A paper read at a meeting of the Bristol Medico-Chirurgical Society, on February 8th, 1899.

As a result of the causes of bronchitis, upon the nature of which I do not intend to dwell, a hyperæmia and swelling of the mucous membrane lining the bronchial tubes take place. This is succeeded after a variable interval by a secretion of mucus from the epithelium and glands. The mucus, at first very thick and tenacious, becomes more watery and gradually diminishes in amount until health is restored. The course of an attack of bronchitis may be correspondingly divided into a first and second stage, the onset of secretion from the mucous membrane marking the division between them. The inflammation of the bronchial mucous membrane may be associated during all its stages with a greater or less degree of spasm of the underlying bronchial muscles.

The beginning of even a mild attack of bronchitis is generally accompanied by a slight elevation of temperature, the patient has a dry cough, feels "tight" or "raw" within his chest, the breathing is more rapid and is accompanied by a sense of oppression. Physical examination of the lungs in this stage reveals nothing, or perhaps a little sibilus and rhonchus.

Now one finds, from observation of the course of the disease, that, as soon as the mucous membrane begins to secrete, the increased rate of breathing and its accompanying difficulty disappear, and the patient feels better. Our efforts then should be directed towards two ends—the reduction of temperature and the speedy induction of secretion.

The patient should obviously be kept in bed until all fever is gone. Antipyretic drugs are certainly not required in the mild cases here dealt with. All that is needed is some sudorific —for instance, sweet spirit of nitre or acetate of ammonia. The latter is more suited for continuous administration, whereas the former if given in a sufficient dose acts more quickly. Two drachms of nitre, mixed with water immediately before administration, will generally produce perspiration in an adult, and can generally be taken by a child of say ten years; some children, however, vomit a dose greater than one drachm.

The induction of secretion by the inflamed mucous membrane can be most speedily brought about by the administration of alkalies. The results of clinical observation agree with physiological observation in showing that alkalies rapidly produce a secretion from the bronchial mucous membrane, or, if there be already a secretion going on, increase the proportion of water and so render the mucus thinner and more easy of expectoration. Direct antacids are to be avoided, as in large doses they may irritate the stomach. Of indirect antacids the best are citrate of potash and acetate of soda, of which the latter is perhaps the preferable, as sodium salts are less poisonous than potassium ones.

Ipecacuanha has an action on mucous secretions very similar to that of alkalies; but it has the defect that adults, unless cyanosed, are sometimes nauseated by even ten minims of the vinum; so that its use is best confined to children.

Antimony, if given in small doses, say seven and a half minims of the vinum, is a most useful drug at the commencement of an attack of bronchitis. If the patient be feverish it acts as a sudorific, and it diminishes the inflammatory hyperæmia of the bronchial mucous membrane. In large doses antimony has a depressing effect, so that even small ones are best withheld if there be any cardiac weakness. If there be any troublesome cough, ʒ ss. to ʒ j. of syrup of tolu is a useful addition to the above drugs. A mustard leaf, too, will quickly relieve "tightness" or "rawness" of the chest and the accompanying irritating cough.

The last point worthy of mention in the treatment of the first stage of bronchitis is the use of antispasmodics in cases where there is spasm of the bronchial muscles. The inconvenience of such drugs as nitro-glycerine and erythrol tetranitrate is that they are apt to produce headache, and of such drugs as nitrite of sodium that their effect is evanescent. But good results may be obtained by the administration of caffeine, the usefulness of which was advocated by Dr. Skerritt some time ago. The only bad effect I have found is that it occasionally prevents sleep.

The antispasmodics act very imperfectly in relieving spasm of the bronchial muscles due to inflammation of the mucous membrane, until secretion has set in. For instance, whilst a combination of alkalies and caffeine will be found to act very well, caffeine by itself will fail to relieve. This absence of effect may be due to the fact that in some cases narrowing of the calibre of the bronchial tubes is the result of the swelling of the mucous membrane; but I doubt if this be the full explanation of the fact.

At the commencement, then, of an attack of subacute bronchitis, good results will generally be found to follow from (1) the administration of a sudorific; (2) the administration of an indirect alkali, such as citrate of potash or acetate of soda, in 20 to 30 grain doses every three or four hours, combined with a small dose of antimony if circumstances permit; (3) the application of a mustard leaf if the chest feel "raw"; and (4) a caffeine pill, of say 5 grains, at night if there be any bronchial spasm. One finds that expectoration is soon established and the patient relieved of his symptoms. He passes on into the second stage more quickly than he does if left untreated, or treated, for instance, with carbonate of ammonia.

The further question then presents itself, whether it be possible to diminish the secretion of mucus from the bronchial mucous membrane more quickly than would take place if the patient were left untreated. Patients are often first seen in this stage, especially in hospital practice. In regard to this, I should first say that as long as expectoration is difficult, or if it become so during treatment, the administration of indirect alkalies is indicated.

In this second stage there may be a large or moderate amount of phlegm coughed up. If there be a large amount of secretion, accompanied with not more cough than is sufficient to get rid of it, no drug probably does so much good as ammonium chloride, in, say, 20-grain doses every four hours. It has, unfortunately, an unpleasant taste; but this may be partially corrected by spirit of chloroform and syrup. Sometimes a large amount of secretion coming up freely is accompanied by a troublesome tickling cough, and in such a case phosphate of codeine (which is freely soluble in water) in gr. $\frac{1}{4}$ to $\frac{1}{3}$ doses, every four hours, will be found most effectual.

But as a rule the amount of expectoration is moderate, and one needs drugs which will act as astringents, diminish and lessen the total secretion from the mucous membrane, whilst preserving the ratio of its constituents one to the other. Many drugs which are employed for this purpose are apt to have a greater effect on the water than on the mucus, the result of which is that the secretion, though less in amount, is more viscid and difficult of expectoration, and so a troublesome cough is set up. Senega and ammonium carbonate are valuable drugs in diminishing bronchial secretion, but occasionally they produce the above-mentioned untoward result. Tincture of Virginian prune, in ʒss. to ʒj. doses, is useful; it reduces the amount of phlegm and also allays any irritating cough. This latter action is probably dependent on the fact that it contains a small amount of hydrocyanic acid. Its astringent effect, however, is not very great. Tincture of hydrastis is a more effectual astringent, in ʒss. to ʒj. doses, and may often with advantage be combined with the former drug. Euphorbia pilulifera has a somewhat limited use. It is useful in diminishing secretion, and has also the advantage of allaying bronchial spasm. Like stramonium and lobelia, it is apt to cause nausea if given in too large a dose. I have used it in spasmodic asthma; but though its effects are often striking, they are liable to wear off in the course of a few weeks. But this is hardly a defect in the case of an attack of bronchitis. It may be given in the form of the tincture, in ♏ 10 to 30 doses.

Yerba santa—the sacred herb of California—has been used by Indians for many years in the treatment of coughs and colds. Though little known in England, experience in its use shows that it is extremely efficacious in the treatment of the second stage of bronchitis; it seems to diminish the watery and mucous constituents of the phlegm proportionately, so that this does not become more difficult of expectoration. The dose is ♏ 15 to 45 of the liquid extract. It forms a somewhat muddy mixture with water, owing to precipitation of the contained gum-resin; but the addition of a little alkali, ammonium carbonate or bicarbonate of soda, for instance, makes it clearer.

Bronchial spasm in the course of the second stage of bronchitis is best treated with caffeine or iodide of potassium.

The above-mentioned drugs, used for the varying conditions which may arise in the course of an attack, will be generally found to give excellent results in ordinary subacute bronchitis.

In the discussion which followed Dr. Edgeworth's paper, Dr. E. H. E. STACK wished to know if Dr. Edgeworth had tried large doses of vinum ipecacuanhæ in cases of bronchitis of the second stage with spasm, as he had found it very useful in doses of about a drachm every hour for a few hours, and then less frequently, without producing either vomiting or depression.—Dr. MARKHAM SKERRITT was glad that Dr. Edgeworth endorsed his recommendation of caffeine in certain conditions of bronchitis. Similarly to its effect in asthma, caffeine relieved the spasmodic element in bronchitis. In the latter disease, the narrowing of tubes, which caused a certain proportion of the dyspnœa and was expressed by the dry *râles* or rhonchi, was due to two factors: first, inflammatory swelling of the lining membrane and adherence of thick mucus to its surface; and, secondly, spasmodic contraction as in true asthma. In bronchitis, caffeine relieved the latter condition, but did not touch the former; and hence the apparent uncertainty of its effect.—Dr. G. PARKER agreed with Dr. Edgeworth as to the great value of vinum antimoniale in vigorous patients, especially children. The syrup of apomorphia and iodide of potassium were also drugs which he had found very useful. The latter, from its power of favouring exudation, had even been recommended for diagnostic purposes in early phthisis, since its use was followed in a day or two by moist sounds where none previously existed, though some lesion was suspected. He asked also for any experience as to the results of giving vinum ipecacuanhæ by a throat spray, instead of in a draught, which had been recently advised.—Dr. WATSON WILLIAMS desired to emphasise the utility of the old-fashioned poultice, not only for the comfort of the patient, but because there is very clear evidence that these applications of moist heat to the surface of the chest might actually shorten and favourably modify the general course of an attack of acute bronchitis. Simple acute bronchitis is almost invariably due to superficial chill, acting reflexly on the bronchi and producing the vascular engorgement, and it was only reasonable to suppose, as experience seemed to prove actually was the case, that the effect of the poulticing was, by reflex action, to lessen the vascular engorgement and relieve any associated bronchial spasm. The sooner this could be done the less pronounced would be all the further changes that ensued on this bronchial vascular engorgement.—Dr. R. ROXBURGH remarked on the great potency of potassium iodide in releasing bronchial spasm, but insisted on large doses as generally requisite. In spasmodic asthma he would give twenty grains, combined with a like quantity of tincture of belladonna and spirit of chloroform, and would repeat the dose in an hour if necessary; while in bronchitis with much spasm he gave rather smaller doses.

